# Persistence of Mortality-Dominant Pancreatitis Burden Despite Declining Rates, 1990–2023: An Analysis of the Global Burden of Disease 2023 Study

**DOI:** 10.3390/medsci14020309

**Published:** 2026-06-12

**Authors:** Arkadeep Dhali, Ali Shan Hafeez, Dushyant Singh Dahiya, Saikat Mandal

**Affiliations:** 1 Academic Unit of Gastroenterology, Sheffield Teaching Hospitals NHS Foundation Trust, Sheffield S57AU, UK; 2 School of Medicine, Dentistry and Biomedical Sciences, Queen’s University Belfast, Belfast BT97BL, UK; 3 Department of General Medicine, CMH Multan Institute of Medical Sciences, Multan 60000, Pakistan; 4 Division of Gastroenterology, Hepatology & Motility, The University of Kansas School of Medicine, Kansas City, MI 66160, USA; 5 NIHR Nottingham Biomedical Research Centre, University of Nottingham and Nottingham University Hospitals NHS Trust, Nottingham NG72UH, UK; 6 Nottingham Digestive Disease Centre, Translational Medical Sciences, School of Medicine, University of Nottingham, Nottingham NG72UH, UK

**Keywords:** Global Burden of Disease, pancreatitis, mortality, morbidity

## Abstract

Background: Whether the fatal and non-fatal composition of aggregate pancreatitis burden has changed over time remains unclear. We assessed long-term changes in the fatal-to-non-fatal composition of aggregate pancreatitis burden using Global Burden of Disease (GBD) 2023 estimates. Methods: We conducted a systematic descriptive and trend analysis using publicly available estimates from the GBD 2023 Results Tool for incidence, prevalence, deaths, years lived with disability (YLDs), years of life lost (YLLs), and disability-adjusted life-years (DALYs) across 204 countries and territories from 1990 to 2023. Because GBD reports pancreatitis as an aggregate cause category, the analysis could not distinguish acute pancreatitis, recurrent acute pancreatitis, chronic pancreatitis, or acute exacerbations of chronic pancreatitis. Primary analyses used age-standardised rates per 100,000 population. Four burden–composition metrics were derived within each location–year stratum: the YLL:YLD ratio, YLD:DALY proportion, deaths-to-incidence ratio, and prevalence-to-incidence ratio. Temporal trends were modelled in R version 4.5, using segmented regression, with up to three joinpoints selected by a Bayesian information criterion. Results: Globally, all six age-standardised native GBD measures declined between 1990 and 2023. The age-standardised incidence rate decreased from 37.62 (95% UI 32.20–43.11) to 32.91 (28.84–37.17) per 100,000, prevalence from 93.78 (69.26–126.25) to 68.92 (52.53–90.32), deaths from 1.76 (1.49–2.16) to 1.40 (1.21–1.66), YLDs from 5.70 (2.75–9.45) to 4.34 (2.18–7.04), YLLs from 55.96 (46.50–69.72) to 43.60 (36.89–53.53), and DALYs from 61.66 (50.62–75.61) to 47.94 (40.57–58.16). However, the fatal-to-non-fatal composition changed little: the global YLL:YLD ratio was 9.82 in 1990 and 10.04 in 2023, while the YLD share of DALYs was 0.092 and 0.091, respectively. Joinpoint modelling showed fluctuation rather than a sustained shift toward disability-dominant burden: the global YLL:YLD ratio was stable until 1998, increased from 1998 to 2002 (annual percent change [APC] 1.38%, 95% CI 0.42 to 2.36), and then declined modestly thereafter (APC −0.13%, −0.20 to −0.06). Burden remained higher in males, whereas females had a greater non-fatal share of total burden (YLD:DALY in 2023: 0.134 vs. 0.073). All sociodemographic index strata remained mortality-dominant in both 1990 and 2023; low-SDI settings had the greatest fatal dominance (YLL:YLD 34.94 in 1990; 24.72 in 2023). Using a descriptive YLD:DALY ≥ 0.50 benchmark, 203 of 204 countries and territories remained below the disability-dominant threshold in both years, no country crossed from below to above this benchmark, and only Georgia moved from above to below the benchmark. Conclusions: Despite declines in global incidence, mortality, and DALY rates, the aggregate GBD pancreatitis burden remained overwhelmingly mortality-dominant from 1990 to 2023. Because GBD pancreatitis combines acute and chronic pancreatitis, this finding should be interpreted as describing the modelled aggregate pancreatitis cause category rather than proving subtype-specific mortality dominance. The intensity of fatal dominance varied by sex, SDI, region, age, and country, but a structural shift toward disability-dominant aggregate burden was not observed.

## 1. Introduction

Pancreatitis remains an important global digestive disease, encompassing acute and chronic pancreatic inflammation and causing substantial morbidity and premature mortality. Previous Global Burden of Disease (GBD)-based analyses show that its burden is heterogeneous across age, sex, geography, and sociodemographic development. In a GBD 2017 analysis across 195 countries and territories, age-standardised prevalence and the rates of years lived with disability (YLDs) increased, whereas age-standardised incidence decreased between 1990 and 2017, highlighting divergent fatal and non-fatal epidemiological patterns [1]. A separate GBD 2019 analysis of acute pancreatitis estimated 2.81 million incident cases, 115,053 deaths, and 3.64 million disability-adjusted life-years (DALYs) globally in 2019, with substantial regional variation despite declines in age-standardised incidence, mortality, and DALY rates [2]. Age–period–cohort modelling further showed that incident cases and deaths increased 1.63- and 1.65-fold from 1990 to 2019, respectively, while age-standardised rates declined, suggesting that demographic change may obscure underlying rate reductions [3]. More recent GBD 2021 analyses similarly reported increasing absolute cases, deaths, and DALYs despite declining age-standardised rates and persistent regional and socioeconomic disparities [4].

However, previous global analyses have largely reported incidence, prevalence, deaths, YLDs, years of life lost (YLLs), and DALYs as parallel measures, without specifically quantifying whether pancreatitis burden is shifting from premature mortality toward disability. This distinction is clinically and policy-relevant because the balance between YLLs and YLDs may reflect survival, chronicity, case-mix, diagnostic ascertainment, coding practice, and health system capacity. Therefore, we used GBD 2023 estimates to evaluate whether the aggregate global burden of pancreatitis showed evidence of a shift in fatal-to-non-fatal composition from 1990 to 2023 across global, regional, national, sex, age, and SDI strata.

## 2. Methods

We conducted a systematic descriptive and trend analysis of the fatal and non-fatal burden of pancreatitis using publicly available estimates from the Global Burden of Disease Study 2023 (GBD 2023) Results Tool [5]. GBD 2023 reports pancreatitis as an aggregate cause category and does not provide separate Results Tool outputs for acute pancreatitis, recurrent acute pancreatitis, chronic pancreatitis, or acute exacerbations of chronic pancreatitis in the data used for this analysis. Consequently, all burden–composition metrics in this study describe the aggregate pancreatitis cause category. GBD provides internally harmonised estimates of incidence, prevalence, deaths, years lived with disability (YLDs), years of life lost (YLLs), and disability-adjusted life-years (DALYs) for 204 countries and territories by year, age, sex, and geography, with age-standardised rates available for cross-population comparisons [6]. GBD cause-specific mortality estimates are generated within the cause-of-death modelling framework using the Cause of Death Ensemble model (CODEm), whereas non-fatal estimates are generated with DisMod-MR 2.1, a Bayesian meta-regression platform that enforces coherence among incidence, prevalence, remission, and excess mortality. Official GBD uncertainty intervals (UIs) are propagated from 1000 draws and are available for native GBD outputs downloaded from the Results Tool [5].

The analytical files retrieved comprised age-standardised rate series for the globe, sex, SDI groups, countries, and a regional file containing 46 regional or supraregional aggregates, together with age pattern data for the global level. For age pattern rankings, aggregate overlapping age categories were excluded, and rankings were restricted to mutually exclusive age groups to avoid comparing parent age categories with their component subgroups. The primary analyses were based on age-standardised rates per 100,000 population. From the six native GBD measures, four investigator-defined burden–composition metrics were derived within each location–year stratum: the YLL:YLD ratio, calculated as the age-standardised YLL rate divided by the age-standardised YLD rate; the YLD:DALY proportion, calculated as the age-standardised YLD rate divided by the age-standardised DALY rate; the deaths-to-incidence ratio, used as a population-level severity proxy; and the prevalence-to-incidence ratio, used as a crude proxy for chronicity or duration. In practical terms, a higher YLL:YLD ratio indicates that premature mortality contributes more strongly than disability to total pancreatitis-related health loss. A higher YLD:DALY proportion indicates that non-fatal morbidity accounts for a larger share of total burden. The deaths-to-incidence and prevalence-to-incidence ratios were used only as descriptive ecological indicators and should not be interpreted as individual-level case fatality or disease duration estimates.

Temporal trend analyses for burden–composition metrics were conducted in R with the *segmented* package. For the YLL:YLD ratio, segmented log-linear models were fitted with calendar year as the independent variable and the natural logarithm of the ratio as the outcome. Values less than or equal to zero were replaced with 1 × 10^−6^ before transformation to avoid computational failure. For the YLD:DALY proportion, segmented linear models were fitted on the untransformed proportion. For each stratum, models with zero to three joinpoints were compared, and the optimal model was selected by the Bayesian information criterion. Segment-specific annual percent changes (APCs) for YLL:YLD were derived as 100 × (e^β^ − 1), where β is the segment slope on the log scale. For YLD:DALY, slopes were reported as annual percentage point changes. Separate models were fitted for the global series, the five SDI groups, males and females, and each regional aggregate included in the supplied regional file. Joinpoints were interpreted as calendar years at which the temporal slope changed materially. Figure generation was based on raw modelled values without smoothing or interpolation. A YLD:DALY threshold of 0.50 was used only as a descriptive benchmark to distinguish burden in which YLDs contributed at least half of DALYs from burden in which YLLs remained the larger component. Values below 0.50 were described as mortality-dominant and values at or above 0.50 as disability-dominant. We recognise that this threshold is not disease-specific and may be stringent for pancreatitis, particularly because the GBD cause category includes acute pancreatitis. Therefore, the country-level results were interpreted alongside continuous absolute changes in YLD:DALY proportion rather than relying only on binary mortality-dominant versus disability-dominant classification. These analyses were descriptive and ecological. They were designed to characterise patterns in modelled population-level GBD estimates.

## 3. Results

**Figure 1:**
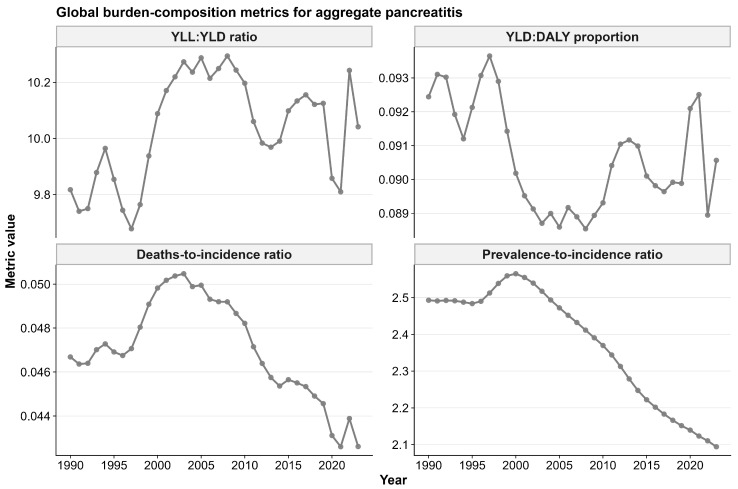
Global temporal trends in derived pancreatitis burden–composition metrics, including YLL:YLD ratio, YLD:DALY proportion, deaths-to-incidence ratio, and prevalence-to-incidence ratio, 1990–2023. Points show annual GBD-derived estimates, and lines show fitted temporal trends. For YLL:YLD ratio, segmented log-linear models were used to estimate annual percent changes; for YLD:DALY proportion, annual percentage point changes were estimated. These derived metrics are point estimates because draw-level uncertainty was unavailable.

**Figure 2:**
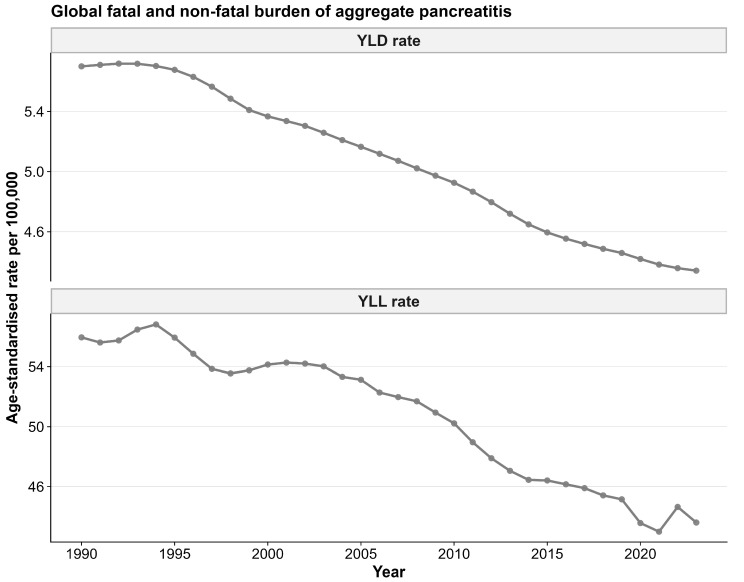
Global age-standardised composition of fatal and non-fatal aggregate pancreatitis burden, showing YLL and YLD rates from 1990 to 2023.

At the global level, all six age-standardised native GBD measures declined between 1990 and 2023. The age-standardised incidence rate decreased from 37.62 (95% UI 32.20–43.11) per 100,000 in 1990 to 32.91 (28.84–37.17) in 2023, prevalence decreased from 93.78 (69.26–126.25) to 68.92 (52.53–90.32), deaths decreased from 1.76 (1.49–2.16) to 1.40 (1.21–1.66), YLDs decreased from 5.70 (2.75–9.45) to 4.34 (2.18–7.04), YLLs decreased from 55.96 (46.50–69.72) to 43.60 (36.89–53.53), and DALYs decreased from 61.66 (50.62–75.61) to 47.94 (40.57–58.16). Despite these declines in native burden measures, the overall fatal-to-non-fatal composition of the aggregate GBD pancreatitis burden changed little. The global YLL:YLD ratio was 9.82 in 1990 and 10.04 in 2023, while the YLD share of DALYs was 0.092 in 1990 and 0.091 in 2023, indicating that the aggregate GBD pancreatitis category remained overwhelmingly mortality-dominant throughout the study period (Figure 1 and Figure 2). The deaths-to-incidence ratio fell modestly from 0.0467 to 0.0426, whereas the prevalence-to-incidence ratio declined from 2.49 to 2.09. These ecological indicators did not provide evidence of a major global shift toward disability-dominant aggregate burden (Figure 1).

Segmented regression showed that the global burden–composition metrics were not monotonic (Figure 1). For the YLL:YLD ratio, the preferred model included two joinpoints, at 1997.9 and 2001.5. The ratio remained stable from 1990 to 1998, with an APC of −0.07% per year (95% CI −0.40 to 0.26); increased from 1998 to 2002 with an APC of 1.38% per year (0.42 to 2.36); and then declined modestly from 2002 onwards with an APC of −0.13% per year (−0.20 to −0.06). For the YLD:DALY proportion, joinpoints occurred at 1997.7 and 2002.1. The proportion was nearly unchanged from 1990 to 1998 at 0.006 percentage points per year (95% CI −0.022 to 0.033), fell between 1998 and 2002 at −0.095 percentage points per year (−0.151 to −0.038), and then rose only minimally thereafter at 0.011 percentage points per year (0.005 to 0.018). Taken together, these models showed fluctuation rather than a sustained structural shift toward disability-dominant aggregate pancreatitis burden.

**Figure 3:**
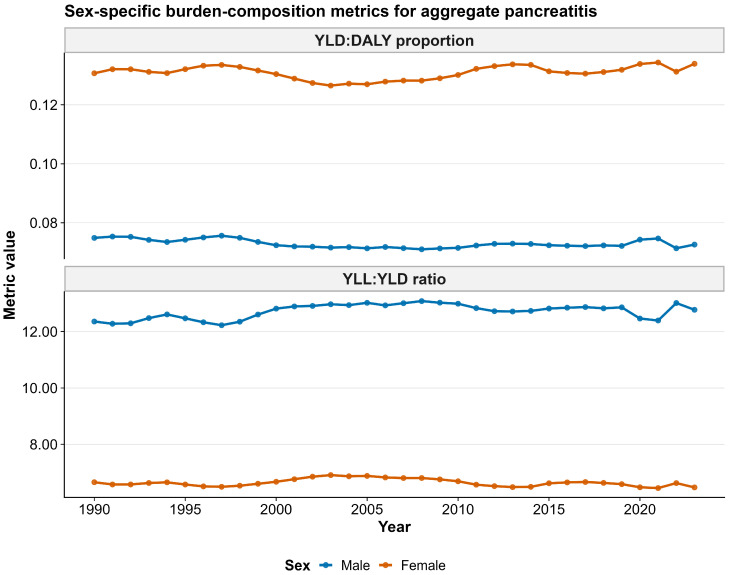
Sex-specific trends in YLD:DALY proportion and YLL:YLD ratio for aggregate pancreatitis among males and females, 1990–2023.

The global burden remained consistently higher in males than in females across the full study period (Figure 3). In 1990, the age-standardised DALY rate was 85.03 (95% UI 65.60–109.78) per 100,000 in males and 38.12 (30.70–47.58) in females; by 2023, these values had fallen to 67.90 (55.73–85.57) and 27.97 (22.21–34.84), respectively. The same pattern was seen for deaths, with male rates decreasing from 2.31 (1.78–3.04) to 1.88 (1.57–2.33) and female rates from 1.22 (0.99–1.55) to 0.93 (0.77–1.16). However, females consistently had a substantially higher non-fatal share of total burden than males. The YLD:DALY proportion was 0.131 in females and 0.075 in males in 1990 and 0.134 and 0.073, respectively, in 2023; conversely, the YLL:YLD ratio was lower in females than in males in both 1990 (6.65 vs. 12.36) and 2023 (6.47 vs. 12.77), indicating that the aggregate pancreatitis burden in women was less mortality-dominant than in men but still far from disability-dominant (Figure 3). Trend modelling supported this sex divergence: in men, the YLL:YLD ratio increased until 2005 at 0.40% per year (95% CI 0.26 to 0.54) and then declined modestly thereafter at −0.14% per year (−0.26 to −0.03), whereas in women the trajectory was more segmented, with a rise from 1998 to 2003 at 1.18% per year (0.71 to 1.64), a decline from 2003 to 2012 at −0.64% per year (−0.93 to −0.34), and near stability after 2012.

**Figure 4:**
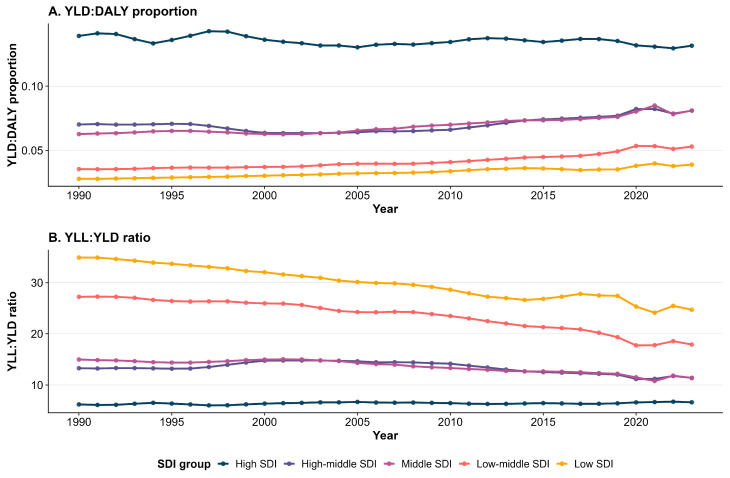
(**A**) SDI-stratified trends in age-standardised YLD:DALY proportion for aggregate pancreatitis across five sociodemographic index groups, 1990–2023. (**B**) SDI-stratified trends in age-standardised YLL:YLD ratio for aggregate pancreatitis across five sociodemographic index groups, 1990–2023.

Across SDI strata, all groups remained mortality-dominant at both time points, but the intensity of mortality dominance varied markedly (Figure 4A,B). In 1990, the YLD:DALY proportion ranged from 0.028 in low-SDI settings to 0.139 in high-SDI settings. By 2023, it ranged from 0.039 in low-SDI settings to 0.132 in high-SDI settings. Correspondingly, the YLL:YLD ratio in 1990 ranged from 6.19 in high-SDI settings to 34.94 in low-SDI settings and in 2023 from 6.60 to 24.72. Thus, although lower-SDI strata showed reductions in fatal dominance over time, they still had a far larger mortality component than higher-SDI strata by the end of the study period. Trend models showed that low-SDI settings underwent sustained declines in YLL:YLD, including APCs of −1.00% per year (95% CI −1.13 to −0.87) before 2008 and −1.87% per year (−2.60 to −1.14) from 2009 to 2014, whereas high-SDI settings showed a more complex oscillating pattern with a brief rise from 1998 to 2005 at 1.23% per year (0.37 to 2.09), a decline from 2005 to 2013 at −0.81% per year (−1.36 to −0.27), and a subsequent increase from 2013 onwards at 0.58% per year (0.25 to 0.93) (Figure 4B). For YLD:DALY, low- and low–middle-SDI groups generally moved upward over time, but none approached the 0.50 threshold used to define disability-dominant burden (Figure 4A).

**Figure 5:**
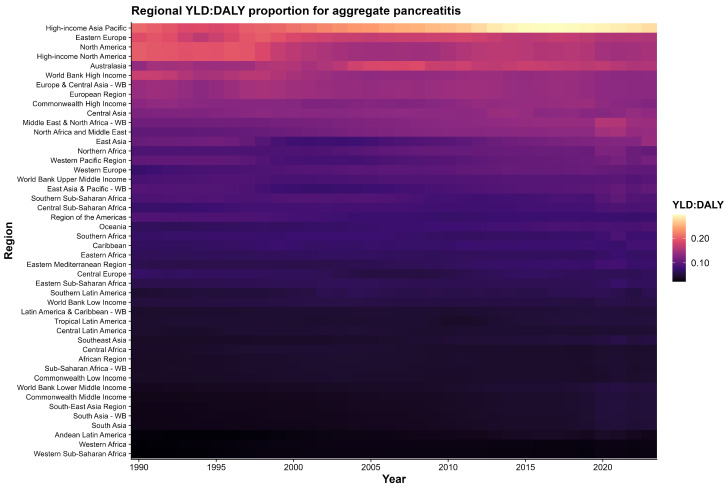
Regional heatmap of age-standardised YLD:DALY proportion for aggregate pancreatitis across regional and supraregional aggregates, 1990–2023.

The regional heatmap showed persistent geographic heterogeneity in non-fatal burden share, with the highest YLD:DALY proportions concentrated in higher-income regions and parts of eastern Europe and the lowest values concentrated in sub-Saharan Africa and parts of south Asia (Figure 5). Among the 46 regional or supraregional aggregates in the supplied regional file, high-income Asia Pacific had the highest YLD:DALY proportion in both 1990 and 2023, rising from 0.205 to 0.277, while western sub-Saharan Africa had the lowest values in both years, increasing only from 0.024 to 0.028. The corresponding YLL:YLD ratios ranged from 3.88 to 41.55 in 1990 and from 2.61 to 34.65 in 2023, underscoring the degree to which fatal dominance remained concentrated in lower-resource settings. These differences were visually stable across the full time series, with only modest upward movement in the non-fatal share in most mortality-heavy regions.

**Figure 6:**
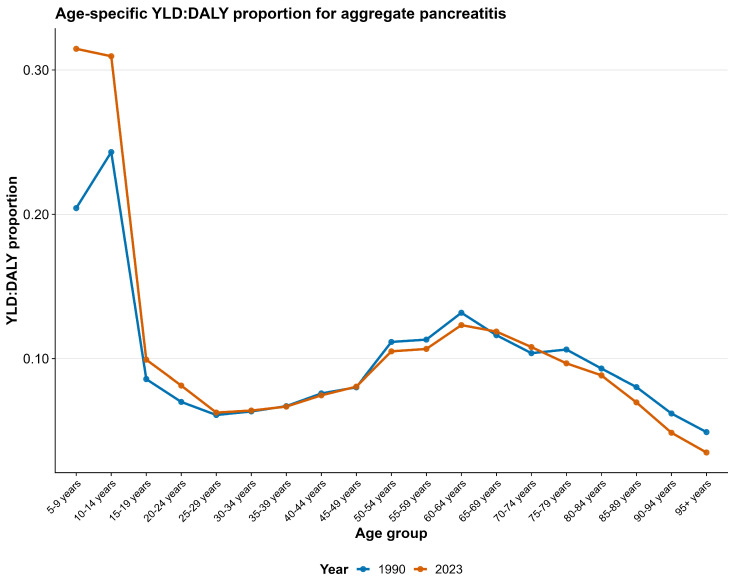
Age-specific YLD:DALY proportion for aggregate pancreatitis in 1990 and 2023, showing changes in non-fatal share of total burden across age groups.

Age pattern analysis showed that the non-fatal share of total burden was the greatest in childhood and early adolescence and the lowest in the oldest age groups (Figure 6). In the mutually exclusive age groups used for ranking, the highest YLD:DALY proportions in 2023 were observed in those aged 5–9 years (0.315) and 10–14 years (0.310), both higher than their 1990 values of 0.204 and 0.243, respectively. By contrast, the lowest 2023 values among mutually exclusive age groups were observed in those aged 95 years or older (0.035) and 90–94 years (0.049). This gradient indicates that, within pancreatitis burden, disability constitutes a larger fraction of total health loss at younger ages, whereas premature mortality dominates increasingly strongly at advanced ages.

**Figure 7:**
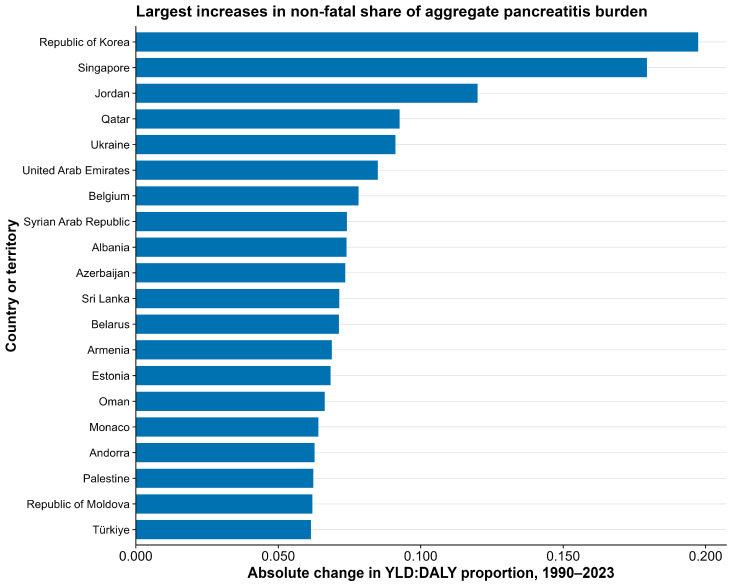
Top 20 countries with largest absolute increase in YLD:DALY proportion for aggregate pancreatitis between 1990 and 2023.

**Figure 8:**
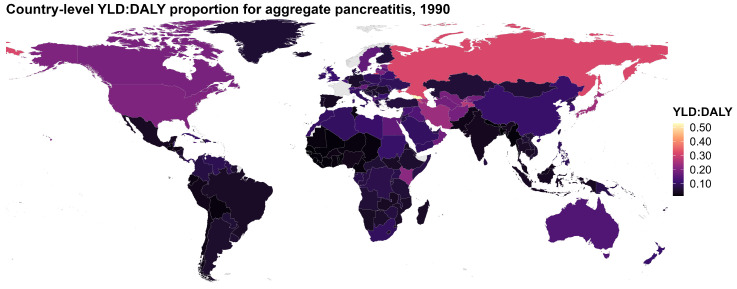
Global distribution of age-standardised YLD:DALY proportion for aggregate pancreatitis in 1990.

**Figure 9:**
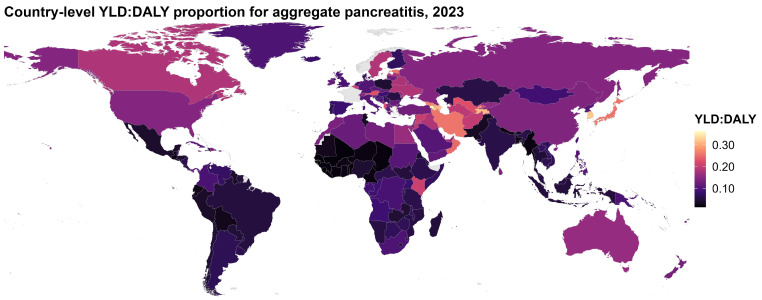
Global distribution of age-standardised YLD:DALY proportion for aggregate pancreatitis in 2023.

**Figure 10:**
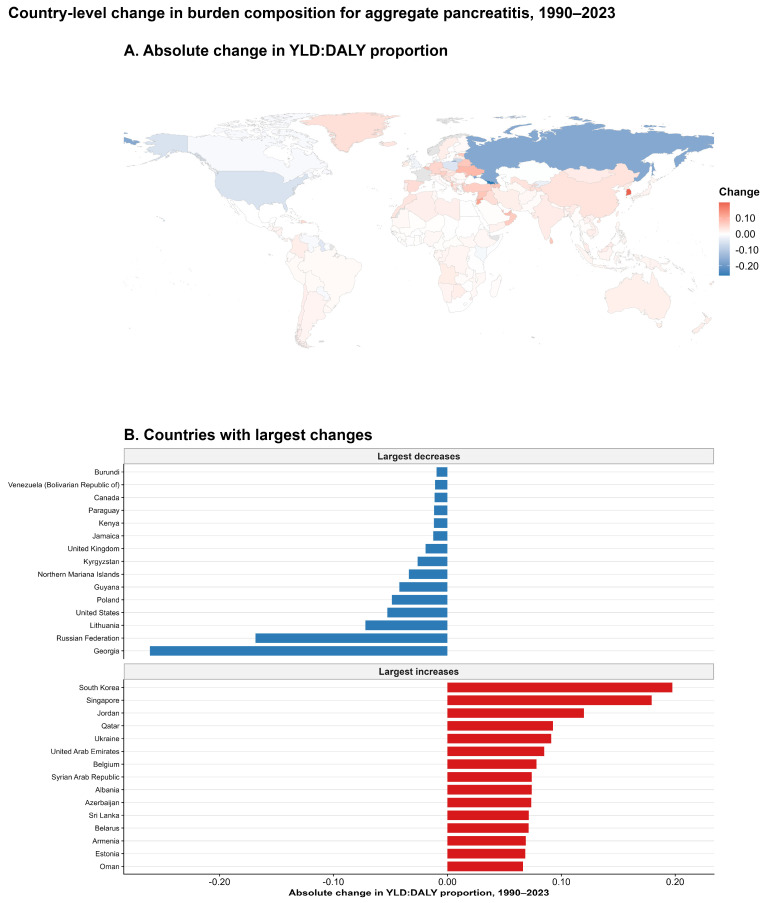
Country-level burden–composition categories for aggregate pancreatitis from 1990 to 2023 based on the descriptive YLD:DALY ≥ 0.50 benchmark and continuous change in the YLD:DALY proportion. The 0.50 benchmark indicates whether YLDs contribute at least half of DALYs and should not be interpreted as a validated disease-specific threshold.

Country-level mapping and ranking showed that crossing the descriptive YLD:DALY ≥ 0.50 benchmark was exceptional rather than typical (Figure 7, Figure 8, Figure 9 and Figure 10). Using the prespecified YLD:DALY ≥ 0.50 descriptive benchmark, 203 of 204 countries and territories remained below the disability-dominant threshold in both 1990 and 2023. Only Georgia was above this threshold in 1990 and below it in 2023, changing from a YLD:DALY proportion of 0.528 to 0.267 (Figure 10). No country crossed from below to above the YLD:DALY ≥ 0.50 benchmark over the study period. However, because this threshold is stringent for an aggregate cause category containing substantial acute fatal burden, these binary classifications should be interpreted alongside continuous changes in YLD:DALY proportion. Nevertheless, several countries recorded sizeable absolute increases in YLD:DALY proportion, led by the Republic of Korea (+0.197), Singapore (+0.179), Jordan (+0.120), Qatar (+0.093), and Ukraine (+0.091) (Figure 7). In 2023, the highest YLD:DALY proportions were observed in Armenia (0.363), Singapore (0.325), the Republic of Korea (0.322), Azerbaijan (0.312), and Tajikistan (0.312), whereas the lowest were in Guinea-Bissau (0.014), Sierra Leone (0.014), Senegal (0.016), Benin (0.017), and Mauritius (0.017) (Figure 8 and Figure 9). These spatial patterns were concordant with the regional heatmap and SDI analyses, again indicating persistent mortality dominance despite heterogeneity in its intensity. Continuous absolute changes in YLD:DALY proportion were therefore more informative than the binary threshold classification for identifying countries with movement toward a larger non-fatal share of aggregate burden.

## 4. Discussion

In this GBD 2023 analysis, age-standardised global pancreatitis burden declined across all six native GBD measures between 1990 and 2023, including incidence, prevalence, deaths, YLDs, YLLs, and DALYs. However, the central finding of this study is that these improvements in conventional burden measures were not accompanied by a meaningful shift in the fatal-to-non-fatal composition of the aggregate GBD pancreatitis burden. The global YLL:YLD ratio remained high, changing only from 9.82 in 1990 to 10.04 in 2023, while the YLD:DALY proportion remained almost unchanged at 0.092 and 0.091, respectively. This suggests that, at the population level, the aggregate GBD pancreatitis category remained overwhelmingly mortality-dominant throughout the study period. These findings complement previous GBD-based studies, which primarily described incidence, prevalence, deaths, and DALYs, by specifically examining the internal structure of pancreatitis burden using fatal-to-non-fatal burden–composition metrics [1,2,3,4,7].

The principal interpretive constraint is that GBD pancreatitis combines acute and chronic pancreatitis. The observed aggregate YLL:YLD ratio may therefore reflect the relative case-mix of acute and chronic disease, the severity distribution of acute pancreatitis, the ascertainment of chronic pancreatitis, and survival after severe attacks, rather than a single intrinsic property of pancreatitis as a unified disease entity. Severe acute pancreatitis can be mortality-dominant because of persistent organ failure, infected necrosis, sepsis, and systemic complications, whereas chronic pancreatitis is typically more disability-dominant because of chronic pain, nutritional impairment, exocrine and endocrine insufficiency, recurrent admissions, unemployment, and impaired quality of life. Therefore, our conclusion of persistent mortality dominance applies to the aggregate GBD pancreatitis category and should not be interpreted as evidence that chronic pancreatitis itself is mortality-dominant.

Our results are broadly consistent with recent GBD 2021 analyses showing declining age-standardised rates despite increasing absolute case numbers. Li et al. reported that global pancreatitis cases increased from 1.73 million in 1990 to 2.75 million in 2021, while the age-standardised incidence rate decreased from 37.62 to 32.81 per 100,000; deaths increased in absolute number, but the age-standardised mortality rate declined from 1.69 to 1.45 per 100,000 [4]. Zhang et al. similarly reported persistent global and regional burden in GBD 2021, with marked sex and geographic heterogeneity and particularly high rates in Eastern Europe [7]. A separate GBD 2019 analysis focused on older adults found substantial pancreatitis burden in older populations, with greater burden among males and persistent cross-national inequalities [8]. Prior European data have shown wide variation in acute pancreatitis incidence across countries, with reported rates ranging from 4.6 to 100 per 100,000 population [9]. Our findings do not contradict these observations; rather, they show that declining age-standardised rates should not be interpreted as evidence of a global transition toward disability-dominant pancreatitis. Instead, the aggregate GBD pancreatitis category remained predominantly driven by premature mortality, even as overall age-standardised rates declined.

The persistence of mortality-dominant aggregate burden is clinically plausible but should be interpreted cautiously. Severe acute pancreatitis can be associated with organ failure, infected necrosis, and other systemic complications, and persistent organ failure has been shown to be strongly associated with fatal outcomes in acute pancreatitis [10]. At the same time, chronic pancreatitis can produce substantial non-fatal morbidity, including chronic pain, disability, unemployment, smoking-associated comorbidity, and impaired quality of life [11]. Therefore, our results should not be read as minimising the disability associated with chronic or recurrent pancreatitis. Rather, they indicate that when pancreatitis burden is aggregated at the population level within GBD, YLLs continue to account for the overwhelming majority of modelled DALYs. This distinction is important because conventional DALY trends may obscure whether improvement is occurring through reduced premature mortality, reduced disability, or both.

The sex-specific findings further support the need to distinguish the amount of burden from the composition of burden. Males had consistently higher age-standardised DALY and death rates than females, consistent with prior GBD 2021 analyses reporting higher ASIR, ASMR, and DALY rates among males [4,7]. However, females had a higher non-fatal share of total burden in our study, with a YLD:DALY proportion of 0.134 in 2023 compared with 0.073 in males. This does not establish a causal sex-specific mechanism, but it suggests that the aggregate male burden was more strongly mortality-dominant, whereas the aggregate female burden had a relatively larger disability component. Prospective cohort data from China have shown that diabetes, gallbladder disease, adiposity, smoking, and heavy alcohol intake were associated with acute pancreatitis risk, with smoking and heavy alcohol drinking being particularly relevant among men [12]. Mendelian randomisation evidence also supports the associations of gallstone disease, smoking initiation, triglycerides, calcium-related traits, and type 2 diabetes with pancreatitis risk, although the strength and specificity of these associations vary by pancreatitis subtype and analytic model [13]. Referral-based data from the North American Pancreatitis Study also support heavy alcohol use and smoking as important risk factors for recurrent acute and chronic pancreatitis [14]. These data provide context for the observed sex differences, but our ecological GBD analysis cannot attribute sex-specific burden directly to individual-level exposures.

Geographic and sociodemographic heterogeneity was substantial. All SDI strata remained mortality-dominant in both 1990 and 2023, but low-SDI settings had a much higher YLL:YLD ratio than high-SDI settings. This pattern is compatible with differences in access to early diagnosis, timely supportive care, intensive care, the management of complications, and the capture of non-fatal disease. However, it may also reflect differences in AP/CP case-mix, diagnostic ascertainment, death certification, and risk factor distributions. These mechanisms cannot be directly separated using summary GBD outputs. Regional differences were also pronounced. High-income Asia Pacific had the highest YLD:DALY proportion in both 1990 and 2023, whereas western sub-Saharan Africa had the lowest. Recent GBD analyses also identified Eastern Europe as a region with high pancreatitis incidence, mortality, and DALY rates [4,7]. In our study, these regional patterns are compatible with roles for health system capacity, population risk profiles, and case ascertainment, all of which may influence not only the level of pancreatitis burden but also whether that burden is expressed predominantly through YLLs or YLDs.

The stronger mortality dominance observed in low-SDI settings may have several explanations. It may represent genuinely higher fatality from severe acute pancreatitis because of delayed presentation, limited access to imaging, intensive care, endoscopic or surgical expertise, and the treatment of complications. However, it may also partly reflect ascertainment differences. In settings with limited diagnostic infrastructure, chronic pancreatitis may be underdiagnosed ante-mortem because diagnosis often requires cross-sectional imaging, endoscopic ultrasound, pancreatic function testing, or specialist evaluation. Conversely, deaths from severe acute pancreatitis may be miscoded as sepsis, shock, diabetic ketoacidosis, or other terminal syndromes when death certification is incomplete. These possibilities mean that the apparent SDI gradient in the YLL:YLD ratio should not be interpreted solely as a difference in biological severity or quality of care.

The age pattern in our analysis also requires careful interpretation. The non-fatal share of burden was the highest in childhood and early adolescence and the lowest in the oldest age groups. This does not mean that younger age groups carry the greatest overall pancreatitis burden; instead, it means that disability accounts for a relatively larger fraction of pancreatitis-related health loss in younger people. Conversely, older age groups had a more mortality-dominant burden profile. These findings are consistent with our observation that fatal burden becomes increasingly prominent at older ages. The contrast between a higher proportional disability component in younger groups and higher mortality dominance in older groups may be useful for planning age-sensitive services, but it should not be overinterpreted as evidence of different disease mechanisms without subtype-specific data.

Risk factor patterns are relevant for interpretation but remain incompletely captured in GBD pancreatitis outputs. Alcohol exposure, smoking, gallstone disease, diabetes, obesity, and hypertriglyceridaemia are established or strongly supported contributors to pancreatitis risk, although their relative importance differs across populations and pancreatitis subtypes [12,13,14]. Translational evidence also supports mechanistic roles for alcohol, fatty acids, bile acids, pathological calcium signalling, mitochondrial injury, premature digestive enzyme activation, endoplasmic reticulum stress, inflammation, and acinar-to-ductal metaplasia in pancreatitis pathogenesis. Alcohol/fatty-acid-related pancreatic injury may also differ from bile-acid-mediated injury in its propensity to progress from acute pancreatitis toward chronic pancreatitis [15]. This mechanistic evidence reinforces the importance of distinguishing pancreatitis subtypes, because aggregate GBD estimates may mask clinically meaningful differences in aetiology, progression, fatality, and disability. At a global level, alcohol remains an important modifiable risk factor. The GBD 2016 alcohol analysis estimated that alcohol was a major contributor to premature mortality and DALYs worldwide, particularly among males [16]. A later GBD 2020 analysis showed that harmful alcohol exposure remained concentrated among younger adults and males, although risk varies by age, sex, and geography [17]. More specifically for pancreatitis, a recent GBD 2021 alcohol-attributable pancreatitis analysis reported increasing absolute deaths and DALYs attributable to alcohol use despite only modest changes in age-standardised rates [18]. These findings are consistent with our observation that age-standardised pancreatitis burden can decline while fatal dominance persists. However, alcohol is not the sole explanation for our findings, and attributing geographic or sex differences entirely to alcohol would be an overstatement.

Metabolic risk is also relevant, particularly when considering long-term trends in pancreatitis burden. The GBD 2021 adult BMI analysis reported the increasing global prevalence of adult overweight and obesity from 1990 to 2021, with continued increases projected under historical trend assumptions [19]. Metabolic risk factors may contribute to gallstone disease, hypertriglyceridaemia, and diabetes-related pancreatitis risk, but the extent to which these drivers explain the YLL:YLD or YLD:DALY patterns in our study cannot be determined from available GBD summary estimates. Similarly, a China-specific GBD 2019 analysis reported declining age-standardised pancreatitis rates but highlighted persistent burden and risk factor heterogeneity at the national level [20]. Taken together, these studies support the idea that pancreatitis prevention should address both alcohol-related and metabolic risk factors while recognising that the contribution of each risk factor is likely to vary by region, sex, and age.

A relevant issue is the relationship between pancreatitis and pancreatic cancer. Both acute and chronic pancreatitis have been associated with increased subsequent pancreatic cancer risk, although interpretation differs by timing, pancreatitis subtype, and potential reverse causation, particularly when pancreatitis occurs shortly before cancer diagnosis [21,22]. This association is not directly captured by the present analysis because GBD assigns pancreatic cancer morbidity and mortality to the pancreatic cancer cause category rather than to pancreatitis. Therefore, pancreatitis-related pathways that culminate in pancreatic cancer may contribute to broader pancreatic disease burden but would not be reflected in the pancreatitis-specific YLLs, YLDs, or DALYs analysed here.

A major strength of this study is that it moves beyond reporting native GBD burden indicators alone and directly examines the balance between fatal and non-fatal burden. The YLL:YLD ratio and YLD:DALY proportion provide a simple, interpretable framework for identifying whether pancreatitis burden is driven mainly by premature mortality or disability. The deaths-to-incidence and prevalence-to-incidence ratios provide additional descriptive context, although they should be interpreted as ecological indicators rather than individual-level measures of severity or chronicity. By applying these metrics across global, SDI, regional, national, sex, and age strata, we found no evidence of a sustained shift toward disability-dominant aggregate pancreatitis burden in the GBD 2023 estimates. Using the prespecified descriptive YLD:DALY ≥ 0.50 benchmark, 203 of 204 countries and territories remained below the disability-dominant threshold in both 1990 and 2023, and no country crossed from below to above this benchmark. Because this threshold is stringent for an aggregate pancreatitis category, the binary classification should be interpreted alongside continuous changes in the YLD:DALY proportion.

This study also has important limitations. First, and most importantly, GBD pancreatitis estimates aggregate acute and chronic pancreatitis. We could not separately assess acute pancreatitis, recurrent acute pancreatitis, chronic pancreatitis, or acute exacerbations of chronic pancreatitis. This is not a minor limitation because these entities differ fundamentally in natural history, fatality, chronic pain, disability, complications, and diagnostic requirements. Therefore, the YLL:YLD ratio in this study may partly represent changing AP/CP case-mix rather than a pure measure of care quality, survival, or intrinsic disease severity. Second, ascertainment and coding differences may influence the central findings. In low-resource settings, limited imaging, endoscopy, specialist access, and death certification may lead to the underdiagnosis of chronic pancreatitis and misclassification of fatal acute pancreatitis as sepsis, shock, diabetic ketoacidosis, or other terminal conditions. These biases could exaggerate or obscure apparent mortality dominance. Third, the derived burden–composition metrics depend on GBD disability weights and modelling assumptions [23]. If chronic pain, pancreatic exocrine insufficiency, endocrine dysfunction, malnutrition, unemployment, psychosocial consequences, and reduced quality of life are incompletely represented in YLD estimates, the non-fatal component of pancreatitis burden may be underestimated. Conversely, changes in disability weights or sequela modelling across GBD rounds could alter the apparent fatal-to-non-fatal composition even if the underlying clinical burden were unchanged. Fourth, the four burden–composition metrics were derived from summary-level GBD estimates rather than posterior draw-level data. Therefore, official uncertainty intervals were reported only for native GBD measures, while derived metrics were treated as point estimates. Fifth, the YLD:DALY threshold of 0.50 was used only as an operational descriptive benchmark and should not be interpreted as a validated clinical threshold. It is particularly stringent for pancreatitis because the GBD category includes acute pancreatitis, which can carry substantial fatal burden. Sixth, GBD estimates depend on data availability, coding accuracy, and modelling assumptions, which vary by country and may be particularly challenging in low-resource settings [6]. Finally, this ecological analysis cannot establish causal relationships between exposures, AP/CP case-mix, health system factors, diagnostic practices, coding quality, and burden–composition metrics.

## 5. Conclusions

Although global age-standardised pancreatitis incidence, prevalence, mortality, YLD, YLL, and DALY rates declined between 1990 and 2023, the aggregate GBD pancreatitis burden remained overwhelmingly mortality-dominant. Because GBD pancreatitis combines acute and chronic pancreatitis, this finding should be interpreted as describing the modelled aggregate pancreatitis cause category rather than proving subtype-specific mortality dominance. The intensity of fatal dominance varied substantially by sex, SDI, region, country, and age, but a structural shift toward disability-dominant aggregate burden was not observed. These findings suggest that global pancreatitis strategies should continue to prioritise the prevention of premature mortality, especially in lower-resource and high-mortality settings, while also strengthening long-term care for survivors, including chronic pain management, nutritional support, and the management of endocrine and exocrine pancreatic insufficiency.

## Data Availability

The data presented in this study are available in the Global Burden of Disease Results Tool at the Institute for Health Metrics and Evaluation, University of Washington: https://vizhub.healthdata.org/gbd-results/ (accessed on 4 April 2026). These data were derived from the following resource available in the public domain: Global Burden of Disease database, Institute for Health Metrics and Evaluation, University of Washington, Seattle, WA, USA. No additional reference number or accession number applies because the analysis used publicly available aggregate query outputs from the GBD Results Tool.
